# Uroperitoneum after Caesarean Section

**DOI:** 10.1155/2013/717623

**Published:** 2013-12-18

**Authors:** Antonakopoulos Nikolaos, Kyritsis Nikolaos, Vlachos Dimitrios, Vlachos Georgios

**Affiliations:** ^1^1st Department of Obstetrics and Gynecology, Athens University Medical School, Alexandra Maternity Hospital, 80 Vasilisis Sofias avenue, 11528 Athens, Greece; ^2^Department of Urology, Alexandra Maternity Hospital, 80 Vasilisis Sofias avenue, 11528 Athens, Greece

## Abstract

Intraoperative injuries of the bladder and the attendant vesicouterine and vesicovaginal fistulas formation are reported as rare events, but the rapid increase in the number of caesarean sections has contributed to the emergence of these complications. Early recognition of these complications makes them much easier to deal with, while simple measures intraoperatively can ensure that these complications will not escape attention. We present our rare case of uroperitoneum after cesarean section, the way of diagnosis and treatment, and due to this incident we review in detail the existing literature on the topic.

## 1. Background

Bladder injury is possible in any standard lower segment caesarean section. This is due to the nature of the operation itself. Following the opening of the abdominal wall, the peritoneum will be incised and the bladder will be pushed downwards, therefore exposing the uterus. The uterus is then cut and the baby is delivered. It is during this movement of the bladder when many bladder injuries occur. Intraoperative bladder puncture during caesarean section is a rare but potentially serious complication, the frequency of which is increased in cases of previous caesarean section or obstetrical hysterectomy [1]. The rapid increase in the number of caesarian sections and therefore repeated caesareans is expected to increase the number of intraoperative complications [2, 3]. Typically bladder injuries are identified intraoperatively. In case of suspected injuries methylene blue infusion into the bladder through urine catheter helps to identify the leak. Cystoscopy is also helpful [1]. The surgical repair should be done early to avoid formation of fistulas, mainly vesicouterine and vesicovaginal [4].

## 2. Case Report

Our case was a 36-year-old G3P3 woman on the 11th day postpartum, after elective caesarean section elsewhere, who was admitted to the emergency department of our hospital because of acute abdominal bloating, decreased urination, and febrile to 38.5°C for 24 hours. The cause of cesarean section was the two previous caesarean sections. The patient's medical history was free of chronic diseases or medications and the only mentioned operations were the three caesareans and an appendectomy in childhood. During the physical examination the patient was afebrile, hemodynamically stable, while the breasts and the trauma of cesarean section did not show any pathology, but there was enough abdominal flatulence, although the abdomen was soft in palpation. The uterine secretions were decreased as expected, serous, not smelly, while the ultrasound scan of internal genitalia was normal. However a large amount of free peritoneal fluid was found. Placed Foley catheter yielded 1800 mL concentrated urine, as in case of proteinuria. The whole clinical figure raised the suspicion of uroperitoneum due to urinary tract injury (bladder and/or ureters) intraoperatively, which was confirmed by aspiration of ascites and biochemical analysis of the fluid. Examination of peritoneal fluid for microorganisms was also negative. Patient was treated conservatively with intravenous hydration, antibiotic coverage with quinolone and metronidazole, and monitoring of urine output through urinary catheter. After urological assessment was conducted, ultrasound of kidney-ureters-bladder and CT urography were ordered, but both failed to highlight the leak. Mostly the above examination ensured the integrity of the upper urinary tract. The following retrograde cystography highlighted a small leak from the upper rear portion of the bladder. The patient underwent laparotomy and after opening the bladder a small deficit of the vesical wall was found with attendant formation of a cavernous pore of 0.5 cm diameter. Both ureteral meatus were normal and functional. Resection of the cavernous pore was conducted and sent for histological examination. Suturing of the bladder in two layers followed, with absorbable sutures 3-0. The postoperative course of the patient was normal and the patient left the hospital on the 7th postoperative day.

## 3. Discussion

Intraoperative bladder injuries, although rarely occurring (0.14 to 0.94%), lead to potentially serious and long-term morbidity [5, 6]. The complications of these injuries are related to prolonged surgical time, urinary tract infections, prolonged urinary catheterization, and prolonged difficulty in mobilizing the patient as well as formation of vesicouterine and vesicovaginal fistulas [7]. The average reported incidence of intraoperative bladder injury is 0.19% in the first caesarean and tripled (0.6%) in previous caesarean section [6].

The first report of vesicouterine fistula in the literature was made in 1908 [8]. Vesicouterine fistulas constitute 1–4% of all urogenital fistulas [9]. The leading obstetrical cause that predisposes their formation is caesarean section, in 83% of cases, while interventional vaginal birth, especially forceps, has been implicated [10–13]. Previous gynecological surgery also predisposes these complications. In particular, urgent caesarean section appears to meet the highest risk and several individual factors have even been studied. The factors found to play a key role were station before surgery (≥+1), low gestational age (<32 weeks), premature rupture of membranes, previous caesarean section, and experience of the surgeon-obstetrician [14, 15].

Moreover, the risk is significantly increased when the caesarean section is carried out at full dilatation, as the operation is much more difficult to perform than an elective caesarean, or a caesarean performed in early labor. Statistics published by the Royal College of Obstetricians and Gynecologists (RCOG) support this viewpoint. Complication rates are greater in caesarean sections performed during labor (24%) than planned procedures (16%). Complication rates are higher at 9 to 10 dilatation (33%) when compared with 0 to 1 cm dilatation (17%). The reason is because, in the advanced stages of labor, the baby's head lies deep in the pelvis. This can make the anatomy appear distorted, making it difficult for the obstetrician to identify where, exactly, an incision should be made. If the bladder is high up over the uterus, then injury is much more likely to occur. In such cases, the damage caused would not be negligent.

The bladder injury is sometimes direct, sometimes is caused by the mobilization of coalesced around tissues, and sometimes is caused by unsuccessful placement of hemostatic sutures [16]. Typically, these bladder injuries are perceived directly intraoperatively. Distal lesions and fistulae creation can result from thermal injury, hematoma, inflammation, ischemia, and prolonged arrest with strong clamp [17]. In suspected lesion dye injection (blue de methylene) into the bladder (or even sterile saline or milk) through a catheter helps identify the outbreak escape. Cystoscopy also helps.

The correction should be made in short time in order to avoid fistulas formation, mainly vesicouterine and vesicovaginal [4]. Also any coexisting infection should be treated preoperatively [18]. Usually, suturing of the bladder wall is made with absorbable suture 3-0 or 2-0, in two layers. The first layer encloses the mucosa of the bladder within the vesical cavity and the next approximates the muscular wall. Foley catheter is usually removed after 7–10 days, but the exact time of catheter removal depends on site, type, and degree of bladder injury [1]. Treatment of fistula is surgical in 95% of cases and conservative treatment requires bladder catheterization for 4–8 weeks and is indicated in fistulas with a good chance of autoregression [19].

## 4. Conclusions

Intraoperative bladder injuries, although rarely occurring, lead to potentially severe and long-term morbidity. The rapid increase in the number of caesarean sections and therefore repeated caesarean is expected to increase the number of intraoperative complications. Intraoperative full wall thickness bladder injuries are easily and immediately understood by the leakage of urine, so treatment is immediate and without late complications. But problem occurs in partial wall thickness injuries that can lead to leakage or fistulas later on, while initially the intact mucosa of the bladder does not allow any leakage of urine, so that the lesion could be seen. Therefore, it is advisable to restore any defect of the muscular coat of the bladder wall to prevent such unpleasant events. Finally, intraoperative urinary catheters with continuous drainage of urine do not always help in the identification of small perforations. In these cases, especially in coexistence of hemorrhagic urine or other suspected lesions, the integrity of the bladder must be checked intraoperatively by filling the bladder with dye (methylene blue) or even sterile saline or milk.
